# Supplementary material for: High-frequency rectifiers based on type-II Dirac fermions
Source: Nat Commun. 2021 Mar 11;12:1584. doi: 10.1038/s41467-021-21906-w (PMC7952558; doi:10.1038/s41467-021-21906-w)
Supplement: Supplementary file 1 — Supplementary Information [file 41467_2021_21906_MOESM1_ESM.pdf]

Supplementary Information

High-frequency rectifiers based on type-II Dirac fermions

Libo Zhang[1,2], Zhiqingzi Chen[1], Kaixuan Zhang[1,2], Lin Wang[1]*, Huang Xu[1], Li Han[1,2], Wanlong Guo[1,3], Yao Yang[1,3], Chia-Nung Kuo[4], Chin Shan Lue[4], Debashis Mondal[5,6], Jun Fuji[5], Ivana Vobornik[5], Barun Ghosh[7], Amit Agarwal[7], Huaizhong Xing[2,8]*, Xiaoshuang Chen[1,3]*, Antonio Politano[9,10]*, Wei Lu[1,3]

[1] State Key Laboratory for Infrared Physics, Shanghai Institute of Technical Physics, Chinese Academy of Sciences, 500 Yu-tian Road, Shanghai 200083, China.

[2] Department of Optoelectronic Science and Engineering, Donghua University, Shanghai 201620, China.

[3] School of Physical Science and Technology, ShanghaiTech University, Shanghai 201210, China.

[4] Department of Physics, National Cheng Kung University, 1 Ta-Hsueh Road, 70101 Tainan, Taiwan.

[5] Consiglio Nazionale delle Ricerche (CNR)- Istituto Officina dei Materiali (IOM), Laboratorio TASC in Area Science, Park S.S. 14 km 163.5 34149 Trieste, Italy.

[6] International Centre for Theoretical Physics (ICTP), Strada Costiera 11, I-34100 Trieste, Italy

[7] Department of Physics, Indian Institute of Technology Kanpur, Kanpur 208016, India.

[8] Shanghai Institute of Intelligent Electronics and Systems, Shanghai 200120, China.

[9] Department of Physical and Chemical Sciences, University of L'Aquila, via Vetoio, 67100 L'Aquila (AQ), Italy.

[10] CNR-IMM Istituto per la Microelettronica e Microsistemi, VIII strada 5, I-95121 Catania, Italy.

**E-mail: wanglin@mail.sitp.ac.cn ; xschen@mail.sitp.ac.cn; xinghz@dhu.edu.cn; antonio.politano@univaq.it.*

Supplementary Note 1: Materials characterization

The superb atomic order with negligible number of defects and the crystalline structure are further confirmed by the analysis of high-resolution transmission electron microscopy (HRTEM) (Supplementary Fig.1a). The spherical-aberration corrected scanning transmission electron microscopy (STEM) in (Fig. 1b), and corresponding selected area electron diffraction (SAED) pattern (inset of Fig. 1b) ensure the single crystal nature. The absence of contamination is confirmed by the analysis of energy dispersive X-ray spectrometry (EDS), as well as the featureless vibrational spectrum and element mapping manifests vibrant color contrast (Supplementary Fig. 1b). Polarization-resolved Raman spectra collected by exciting the fabricated samples by varying the polarization angle with a 532nm excitation laser in Fig.1c and in Supplementary Fig. 1c: peak is found at 80 cm^{-1}, corresponding to the E_g vibrational modes.

Supplementary Note 2: Density functional theory

We performed all the density functional theory (DFT)-based ab initio electronic structure calculations with the projector-augmented-wave (PAW) pseudopotentials and a plane wave basis set using the VASP package. The exchange correlation part of the potential was treated within the generalized gradient (GGA) approximation framework developed by Perdew-Burke-Ernzerhof (PBE). We used 500 eV energy cutoff for the planewave basis set. A k-grid of 12×12×8 is used for the momentum space integration. The electronic optimizations were done within a tolerance of 10^{-7}eV. We relaxed the cell parameters and the atom positions until the residual force

on each atom becomes less than 0.001 eV/Å. The relaxed lattice parameters (a= 3.850 Å and c= 3.260 Å) match very well with the experimental lattice parameters (a= 3.855 Å and c= 5.266 Å). Surface energy spectrum was obtained within the iterative Green's function method as implemented in the WannierTools package.

Supplementary Note 3: The evolution of the Te 5*p* orbitals

Here we briefly describe the evolution of the Te 5*p* orbital manifold, which gives rise to the series of band inversions in $NiTe_2$ and is shown in Fig. 4 of the Supplementary. Step one: the generation of bonding and anti-bonding states due to the strong intra-layer hybridization between the Te^1 and Te^2 orbitals. Step two: the Te^1 and Te^2 orbitals are further split due to a strong trigonal crystal field generated by the layered crystal structure of $NiTe_2$, separating p_z from the p_x, p_y orbitals. Step three: inclusion of spin-orbit coupling further splits the orbitals into $|\boldsymbol{J}, |\boldsymbol{m}_J|\rangle$ states. Step four: highlighting the effect of the dispersion along the Γ - A direction, and the formation of the bulk type-II Dirac point along with multiple band-inversions in the valance band (Supplementary Fig. 5).

The irreducible representation of some of these states at the at Γ and A points and along the Γ - A direction is shown in Fig. 3(b) of the main text. The Δ_4 and $\Delta_{5\text{-}6}$ states along the Γ - A direction generate the bulk Dirac point (BDP). The doubly degenerate Δ_4 and $\Delta_{5\text{-}6}$ bands have opposite rotation characters (+1 and -1, respectively), and due to the C_3 symmetry, the BDP is protected from any gap opening due to perturbations. Originating from the 5*p*-orbital manifold of the Te atoms, the formation of non-trivial band morphology in $NiTe_2$ has a of great significance, in which the BDP and the

series of IBG in $NiTe_2$ give rise to topological spin-polarized surface states over a wide range of energies, including those in vicinity of the Fermi surface.

Supplementary Note 4: Noise current measurement experiment

The noise current of the $NiTe_2$ device is shown in Supplementary Fig. 2e. For noise current density analysis, the noise power spectra are measured using amplifiers and then digitized with a dynamic signal analyzer-SR785. Throughout the noise testing process, our devices are always placed in the opaque shielding box. We calculate the NEP, expressed by NEP = I_n / R_A, where I_n is the noise current density in A / $Hz^{1/2}$. For evaluating NEP values, the noise current is extracted from the noise power spectra at frequency bandwidth of 1 Hz, and the responsivity are estimated at the same frequency.

Supplementary Note 5: Low temperature device performance measurement

We also perform the photocurrent measurement at variable temperatures by using clean and low-disorder sample. As shown in Supplementary Fig. 6a, the resistance of samples decreases with decreasing temperature, as expected. However, we found that the magnitude of the zero-bias current did not increase with the decrease of temperature. In contrast, the current decreases with decreasing temperature, as shown in the Supplementary Fig. 6b. We note that the device has higher photocurrent at low temperature than at room temperature for the case of applying a bias voltage.

The photo-thermoelectric (PTE) effect arises from the local nonequilibrium and it produces an electron-temperature driven photocurrent in the form of $V_T = (S_D - S_S)\cdot \triangle T$, where S_D and S_S are the Seebeck coefficients at two metal-material interfaces. The

Seebeck coefficient can be expressed in terms of the conductivity via the Mott relation[1]:

$$S = \frac{\pi^2 k_B^2 T}{3e} \frac{d \ln(\sigma(E))}{dE} \Big| E = E_f \qquad (1)$$

The trend of the photocurrent from experimental data is clearly different from the trend of photo-thermal response as depicted in Supplementary Fig. 6c, ruling out the PTE dominated mechanism at zero bias.

In the case of device under electrical bias, the resistivity change of a temperature-sensitive material due to the uniform light-heating effect induced by photon absorption[2,3] can also lead to photoresponse. In order to clarify the working mechanism under the bias mode (photoconductive effect and bolometric effect), we extract the photocurrent at different bias voltage and different input power in Supplementary Fig. 4b-c, in which photocurrent can be viewed as $I_P = I_{light} - I_{dark}$ (I_{dark} is the dark current without radiation). The decrease of resistance of the material under strike by terahertz light radiation contradicts the increase of resistance when the material is heating up during temperature-dependent experiment in Supplementary Fig. 6a and Fig. 4a. So, the bolometric effect does not hold when the device is electrically biased. The whole process is explained by the data under zero bias, 10 mV and 100 mV bias in Supplementary Fig. 4g. In self-powered mode, as discussed above the increase of temperature leads to the increase of scattering probability, but by applying a bias greater than 10 mV, we find that the trend of temperature-dependent photocurrent is different from the that of self-powered mode and it can be decomposed as the photoconduction superimposed on the zero-biased response. We

still hold that it is a second-order nonlinear photoresponse even under direct electric field E_{DC} (Supplementary Fig. 4e), the process of which is that the nonequilibrium electrons under electrical bias are accelerated unilaterally from one side to another side of channel due to the bias-induced asymmetry, resulting in a photoconduction behavior. The photogenerated excess carrier density Δn increases the conductance by $\Delta\sigma = \Delta ne\mu$, where μ is the mobility of the channel materials and increases at lower temperatures, since the light absorption rate of the material is significantly enhanced at lower temperatures as shown in supplementary Fig. 4f.

Actually, finite temperature could indeed affect the intraband second-order photo-response through thermally smeared Fermi distribution function and the change of scattering times. It is reasonable from theoretical point of view that the zero-bias rectification will grow up for highly doped semiconductors or semimetals at lower temperature, because of larger momentum relaxation time[4-6] of carrier transport or higher photon absorption.

In the specific realistic case of our system, it can be inferred from temperature-dependent output characteristic that the resistance has been changed by less than 10%, indicating the minimum change of material's transport property at lower temperature. On the other hand, the rectification current depends on the ratio (τ_s / τ_a) between symmetric scattering-time τ_s and asymmetry scattering-time τ_a (e. g. skew-scattering). Accordingly, it is possible that asymmetric scattering time is reduced at higher temperature as related to the skew scattering, leading to the growth of rectification efficiency. It means that, in our specific case, the change of τ_a

dominates the second-order photocurrent.

Anyway, it is desirable that higher mobility could imply superior rectification efficiency. Materials with low carrier density represents promising candidates for such purposes. Thus, it seems that thermally smeared distribution will not degrade significantly the zero-bias rectification current.

Supplementary Note 6: Theoretical basis of anisotropic trigonal crystal-field scattering

To analyze the second-order photoresponse in $NiTe_2$, we turn to a microscopic interpretation by Boltzmann kinetic equation for the carrier distribution function $f_k(\varepsilon)$ under a spatially homogeneous electric field **E**,

$$\frac{\partial f_k}{\partial t} + e\boldsymbol{E}\frac{\partial f_k}{\partial \boldsymbol{k}} = \Upsilon[f_k] \qquad (2)$$

in which $e < 0$ denotes the charge of the electrons and $\Upsilon[f_k]$ is the collision integral, describing the new balance of the carrier acceleration and relaxation due to the scattering processes.

In $NiTe_2$ the spin momentum locked surface charge carriers break the inversion symmetry. In such inversion symmetry breaking surface state, the possibility of skew scattering gives rise to the rectification current. Mathematically, this is expressed by asymmetric transition rates between the k-k' states and the -k and -k' states, $W_{k\,k'} \neq W_{-k,-k'}$. $W_{k\,k'}$ is the transition probability of a carrier with momenta k to momentum k' after scattering. It includes the symmetric ($W^s_{kk'}$) and the asymmetric ($W^a_{kk'}$) transition rates, $W_{k\,k'} = W^a_{kk'} + W^s_{kk'}$ [5,7]. The asymmetric W^a is finite only in systems with inversion

symmetry breaking, and it results in the skew scattering of chiral bloch electrons which give rise to an effective D.C. rectification current [8-10]. parts. The photocurrent can be calculated from the equation,

$$j = e \sum_k v_k f_k \qquad (3)$$

where v_k is the electron's group velocity with momentum k. Solving for the current, by considering the crystal symmetries, along with the related Lorentz force of the radiation magnetic field to solve Boltzmann kinetic equation[11,12], it can be shown that j ~ E_x^2-E_y^2.

Supplementary Note 7: the discussion of side jump

Based on the experimental observations, a contribution arising from the side jump process cannot by unambiguously excluded. However, we note that a finite side-jump contribution generally arises due to the interplay of Berry curvature and scattering, and $NiTe_2$ being a Dirac semimetal does not have a finite Berry curvature in the bulk. Thus, from a theoretical perspective, the role of the side-jump phenomena contributing to the observed rectification current, seems unlikely.

The distribution function $f_k(\varepsilon)$ in nonequilibrium could be decomposed as: $f_k = f_0 + f_k^{ad} + f_k^{sc}$, where f_0 is the Fermi-Dirac distribution, f_k^{ad} is the anomalous distribution function owing to finite potential energy shift and the Berry curvature, and f_k^{sc} is the scattering distribution function with zero potential energy shift, i.e., without the Berry curvature[6,13]. The electron's group velocity v_k is given by $v_k \equiv v_0 + v_{av} + v_{sj}$, where v_0 is the band dispersion velocity unrelated to the Berry curvature, v_{av} is the anomalous velocity connected with the Berry curvature, and v_{sj} is the side-jump velocity[14,15].

From the distribution function, the photocurrent can be calculated from the equation [16],

$$j = e\sum_k v_k f_k = e\sum_k (v_0 + v_{av} + v_{sj})(f_0 + f^{ad} + f^{sc}) \quad (4)$$

As a Dirac semimetal $NiTe_2$, all Berry curvature related functions are neglected. In the weak impurity limit (scattering time $\tau \rightarrow \infty$), the skew scattering contribution ($v_0 f^{sc} \propto \tau^2$) is dominant.

Supplementary Note 8: the Dirac point close to the Fermi surface

There are two possible mechanisms that can give rise to the rectification current. Both of them rely on two different aspects of the $NiTe_2$ electronic structure:

1) The presence of the type-II Dirac cone in nearness of the Fermi energy

   Bulk Dirac fermions are involved in the non-equilibrium carriers, due to the strong electromagnetic fields in the device geometry (see Fig. 2a of the manuscript). It can give rise to the rectification current.

2) The presence of topological surface states near the Fermi energy

   Topological surface states in $NiTe_2$ arise from a band inversion in the conduction region. They can give rise to the rectification current, by means of the chiral Bloch scattering, as discussed in the manuscript.

While we have ruled out several other possibilities for the rectification current, we believe that a combination of both (1) and (2) is responsible for the observation of rectification current in our experiments.

To highlight the importance of the type-II Dirac fermions in $NiTe_2$, we note that in group X Pd- and Pt- based dichalcogenides, the bulk Dirac node lies deep below the Fermi level (~0.6, ~0.8 and ~1.2 eV in $PdTe_2$, $PtTe_2$, and $PtSe_2$, respectively)[17], hindering their successful exploitations in technological applications related to photons with wavelength in the range of microwaves, Terahertz (THz) and far- and middle-infrared (FIR/MIR). In contrast, $NiTe_2$ hosts type-II Dirac fermions in nearness of the Fermi energy, as evident from Fig.1f of the main text. Our spin-resolved ARPES measurements explicitly demonstrate the existence of a pair of

type-II Dirac nodes in $NiTe_2$ along the C_3 rotation axis, lying in the nearness of the Fermi energy.

This establishes $NiTe_2$ as a prime candidate for exploration of Dirac fermiology, with further possible applications (beyond those in our manuscript) in spintronic devices and ultrafast optoelectronics.

In Fig. S10, we provide a comparison of band structures of MTe_2 (with M=Ni, Pd, Pt).

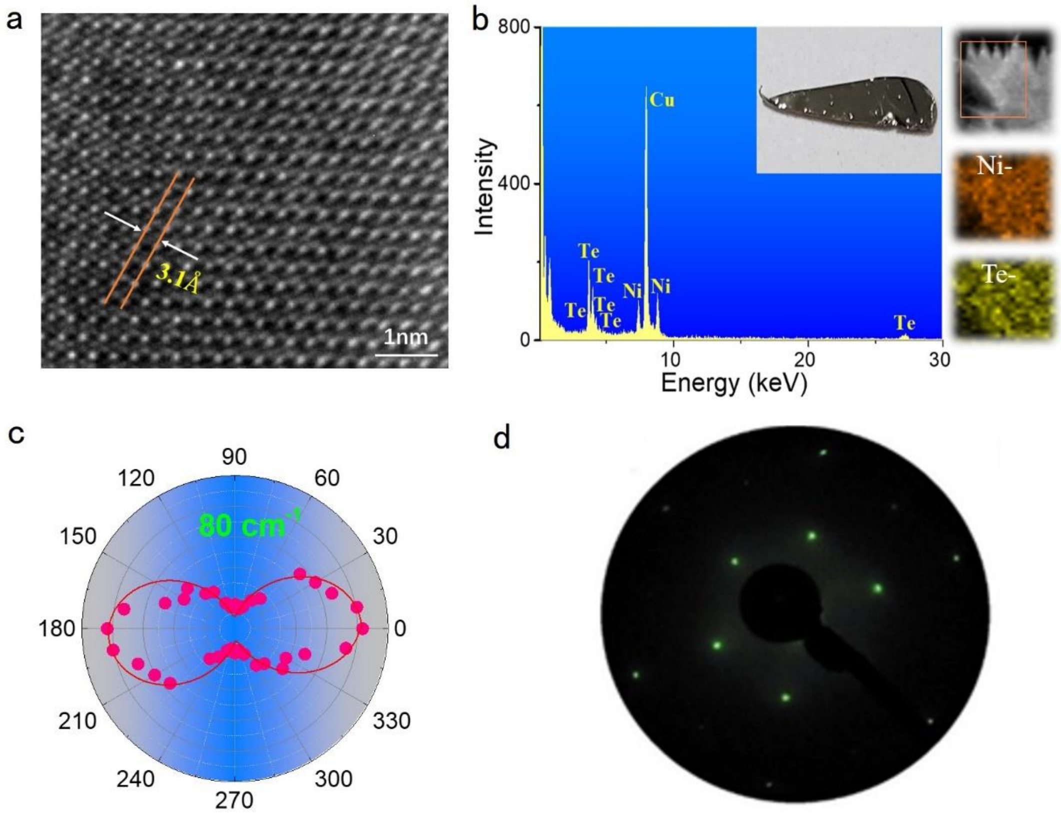

Supplementary Figure 1. a HRTEM of the hexagonal $NiTe_2$ flakes, scale bar of 1 nm. **b** EDS of the $NiTe_2$ flakes, with the photo of grown single crystals in the inset. The signal of Cu element comes from the supporting TEM grid. Right parts show the mapping of Ni and Te elements. **c** Polar figure of the Raman peak intensity of $NiTe_2$ single crystal corresponding to E_g mode. **d** The LEED pattern of the (0001)-oriented single crystals of $NiTe_2$ (acquired at 84 eV of primary electron beam energy).

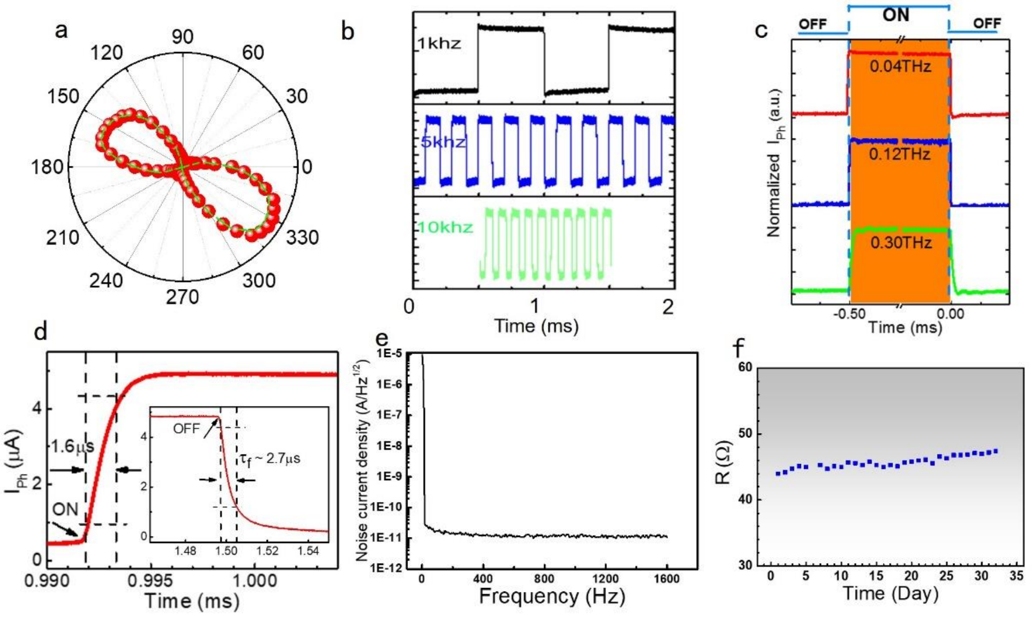

Supplementary Figure 2. a Varying photocurrent at different polarization-angle. Note that the rectification current follows a cos(2θ+φ), where θ is the polarization angle and φ = -30 degrees is the orientation of the crystal axes with respect to the device geometry. This is consistent with the relation $j{\sim}E_x^2-E_y^2$, in terms of the crystal coordinate axes. **b** Time-dependent photocurrent measured for different modulation frequencies of 1khz, 5khz and 10khz. **c** The time-resolved photocurrent response at different frequencies of the optical excitation when the devices work in the self-powered mode, i.e., in a zero-bias condition. **d** Rise and decay (inset) curve of the time-resolved response in a single cycle, showing the rising and decay time of 1.6 μs and 2.7 μs, respectively. The response time is usually defined as the time required by the photoresponse to rise from 10% to 90% or to fall from 90% to 10% in a single impulse. **e** Noise current density as a function of modulation frequency for the $NiTe_2$ device at zero bias using amplifiers and a dynamic signal analyzer-SR785. **f** The change in the resistance of $NiTe_2$ on being exposed to air for about a month. Clearly, the stability is very good and promising for device applications.

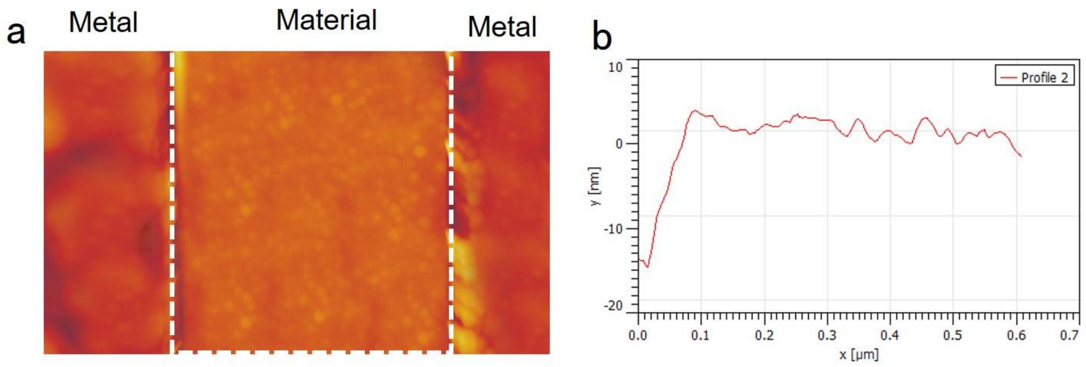

Supplementary Figure 3. a Morphology characterization of the channel region by AFM. **b** The flatness of the sample along the width of the material. The sample thickness fluctuates within 2 nm, with good flatness.

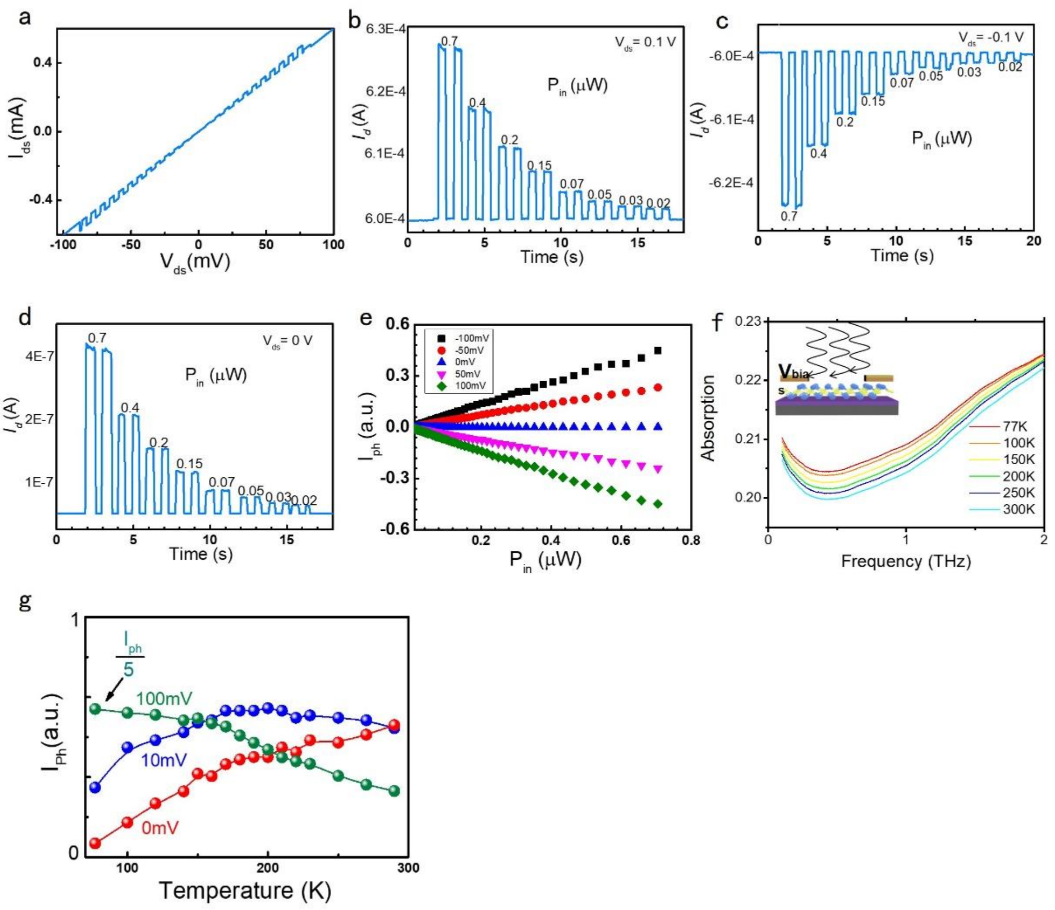

Supplementary Figure 4. a Photocurrent measurement without and with applied bias voltage. **b-d** Dynamic response curve under different high-frequency electromagnetic powers when the device is fixed at certain voltages. **e** Dependence of the photocurrent on the radiation power intensity at different bias voltages. **f** Absorption rate of terahertz radiation at different temperatures. **g** Dependence of the photocurrent on the temperature. The different colored dots represent the photocurrent corresponding to different bias voltages.

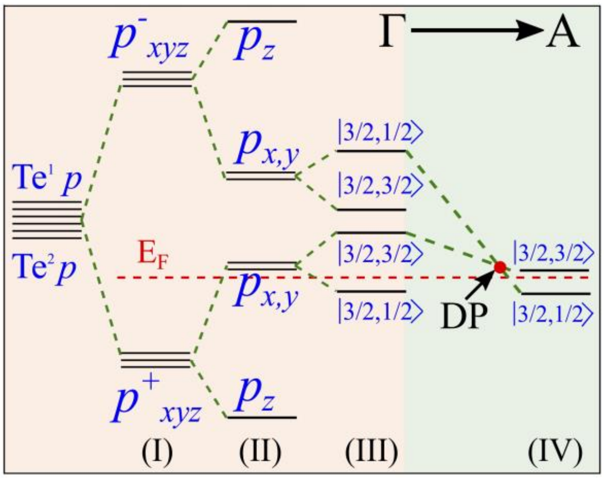

Supplementary Figure 5. The evolution of the Te 5*p* orbitals in the formation of Dirac-cone states in $NiTe_2$. Step (I) shows the the creation of bonding and anti-bonding orbitals. Step (II) shows the effect of the strong trigonal crystal field which separates the p_z orbitals from the $p_{x;y}$ orbitals. In step (III), we show the splitting of these states into the $|\boldsymbol{J}, |\boldsymbol{m}_J|\rangle$ states in the presence of SOC. In step (IV), we demonstrate the effect of out of plane dispersion and the formation of the Dirac point.

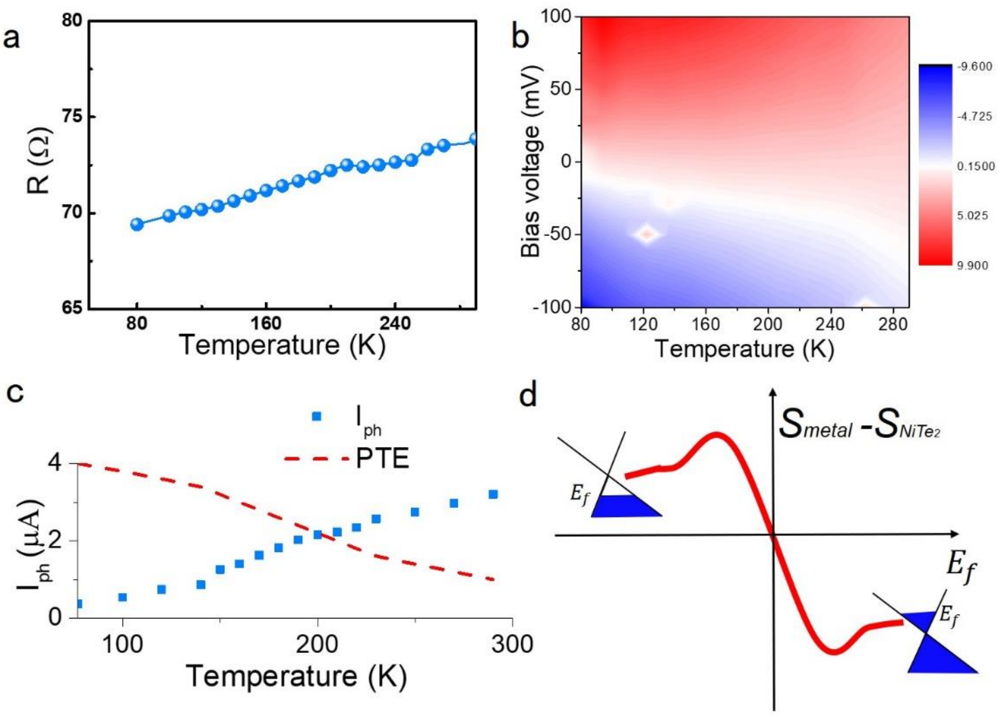

Supplementary Figure 6. a Low temperature measurement based on $NiTe_2$ photodetector. **b** Photoresponse mapping as a function of bias voltage and temperature. **c** Two-terminal photocurrent as a function of temperature (T) without bias voltage. Blue dots: experimental data. Red dots: Theoretical trends of photothermal current at different temperatures. **d** Seebeck coefficient described as a function of E_f[18]

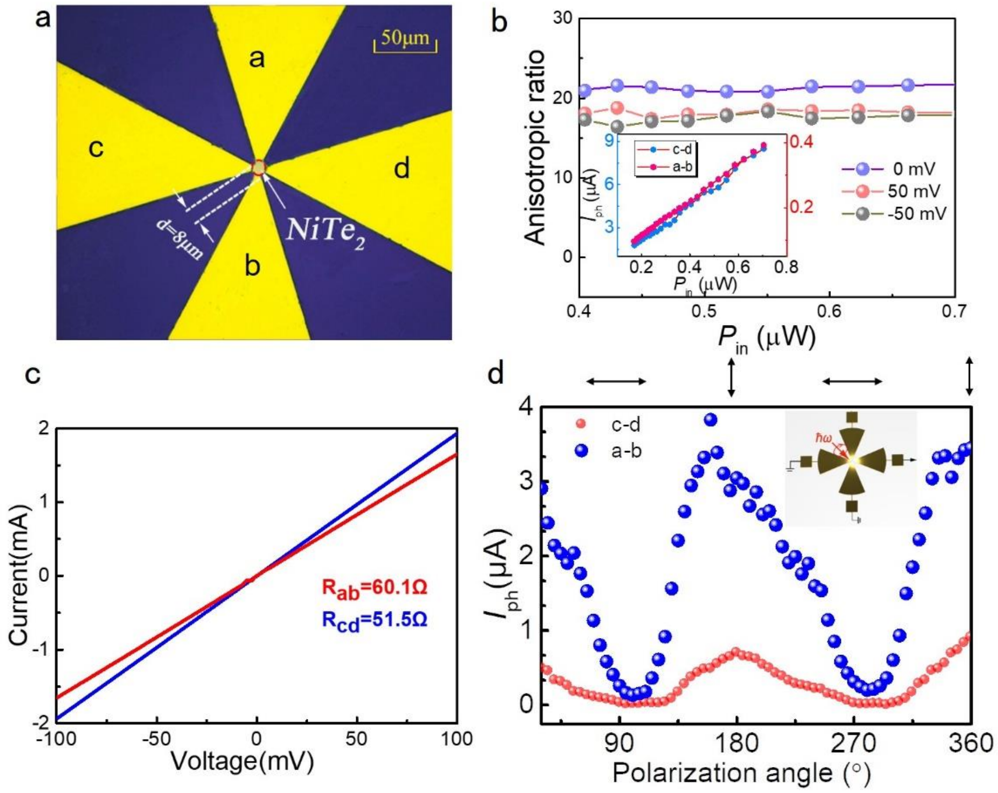

Supplementary Figue 7. Measurement results of the additional device with four-sector antenna. a Optical micrograph of the four-terminal sector antenna. **b** The anisotropic photocurrent response of device along a-b and c-d directions with different input-power. The inset is photoresponse applying zero bias voltage along a-b and c-d directions with P_{in} added up to 0.7 μW (a, b, c, d corresponds to different electrodes as shown in Fig.7a.). **c** Different resistances corresponding to a-b and c-d directions, respectively. **d** Polarization-dependent photocurrent shows the good repeatability of our experimental results.

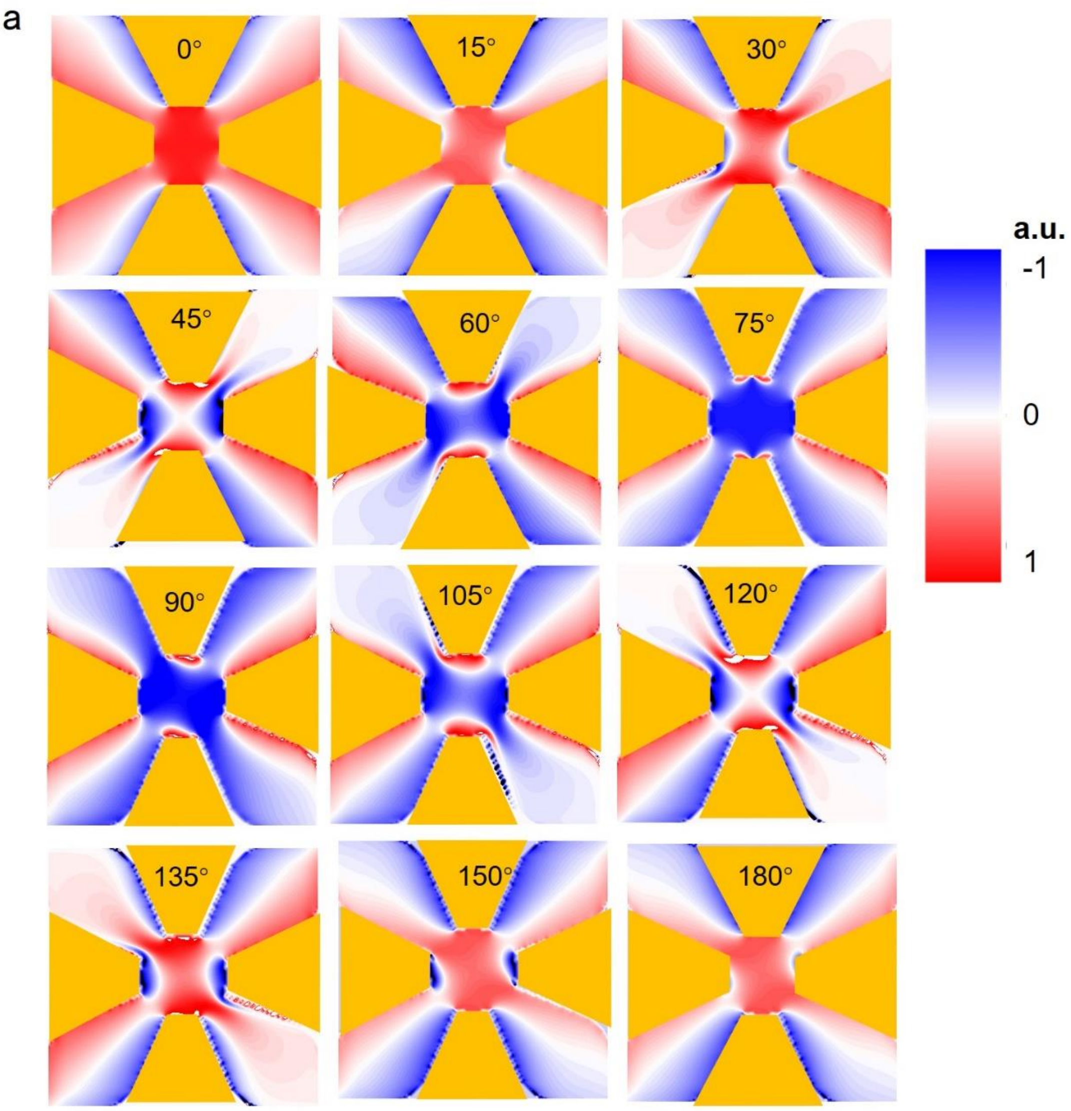

a
0°
15°
30°
45°
60°
75°
90°
105°
120°
135°
150°
180°
a.u.
-1
0
1

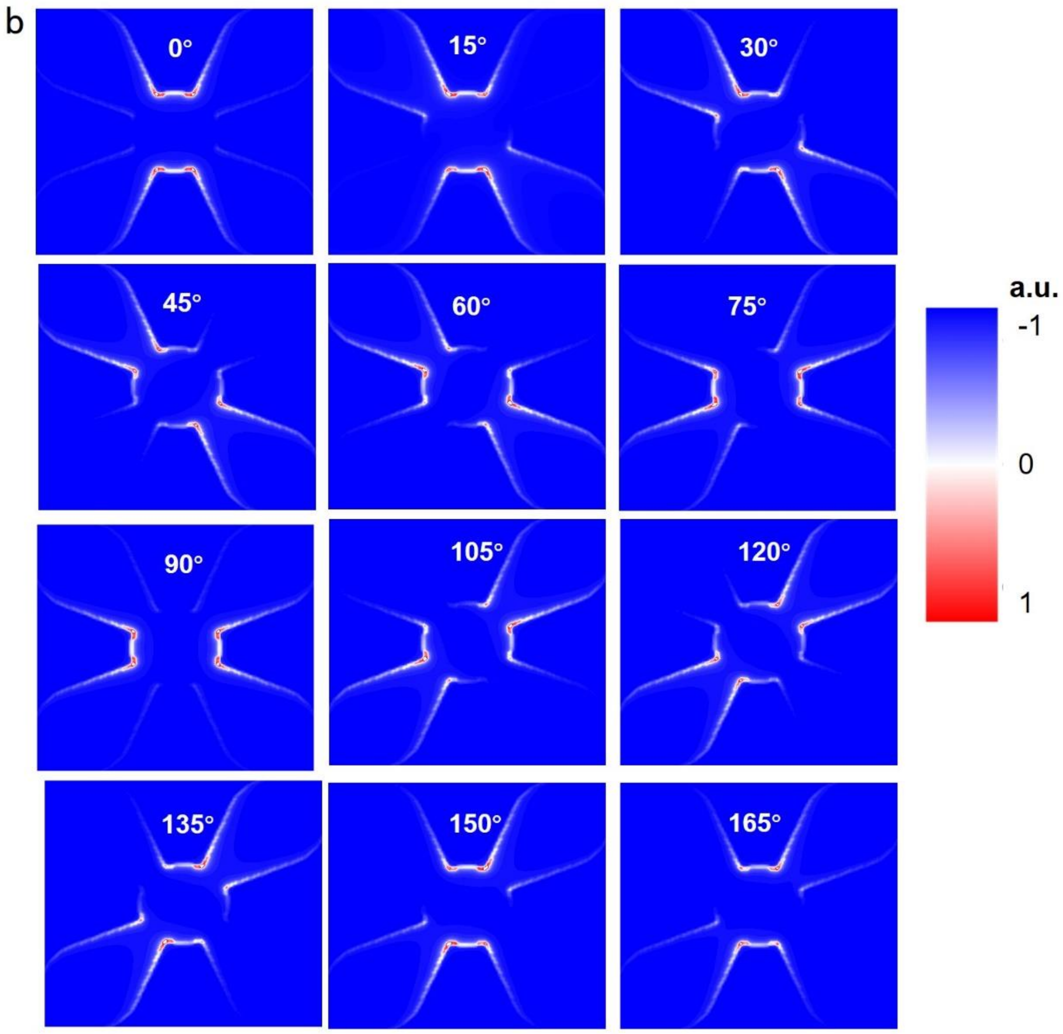

Supplementary Figure 8. a Simulated skew scattering photocurrent distribution at 15 degrees of polarization angle. **b** Simulated the PTE photocurrent distribution at 15 degrees of polarization angle.

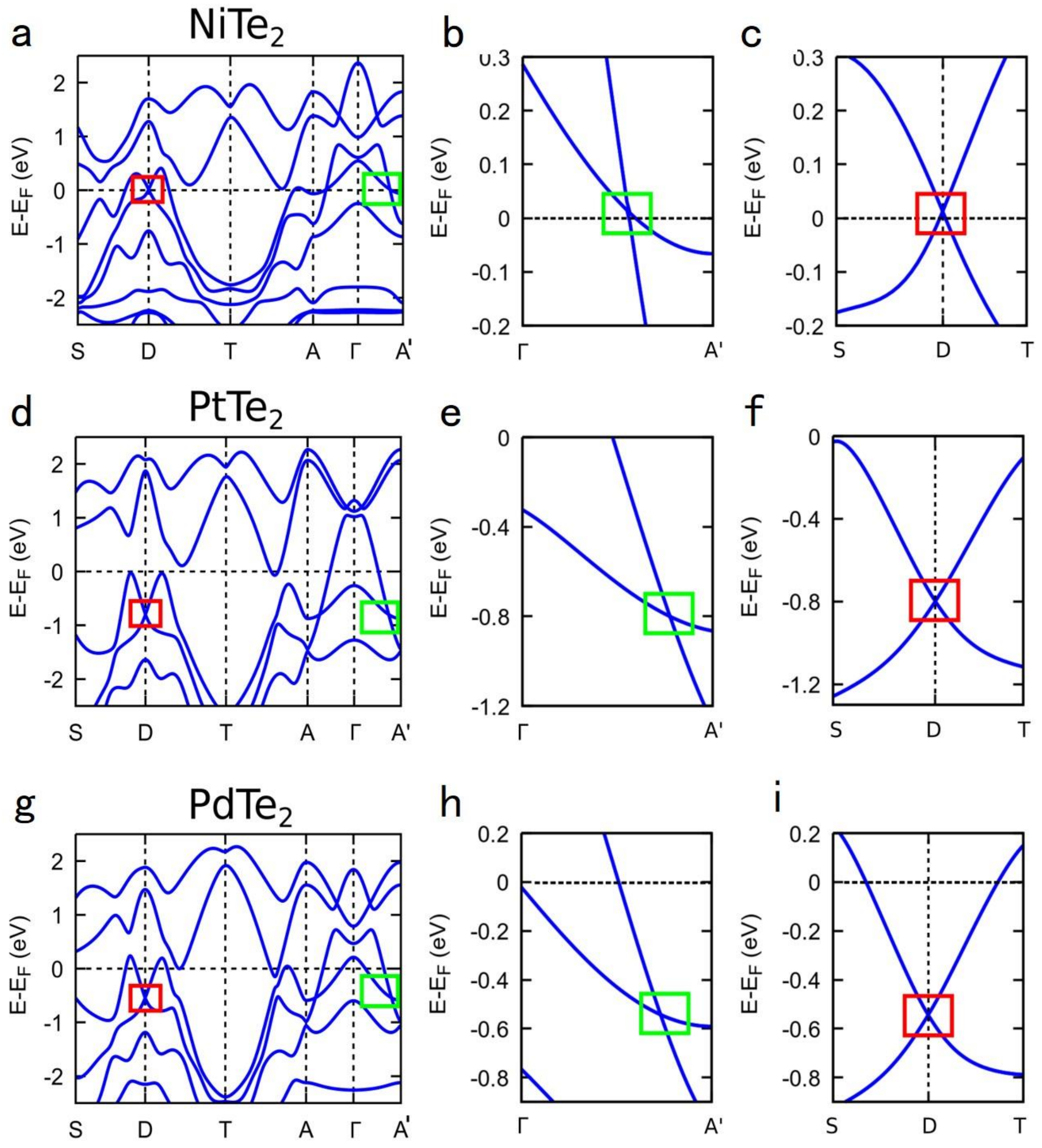

Supplementary Figure 9. The band structure of $NiTe_2$, $PdTe_2$, and $PtTe_2$, showing the two Dirac points tilted in opposite directions, located on the A-Γ-A′ axis. The dispersion around each of the Dirac points is isotropic in the horizontal S-D-T plane (parallel to the experimental Γ-K-M plane) and anisotropic and "tilted" along the Γ − A direction. A magnification of the band structure around one of the Dirac points is shown in panels (a), (f) and (i) for the isotropic S-D-T direction [marked by the red circle], and for anisotropic and "tilted" the Γ – A′ direction in (b), (e) and (h) [marked by the green circle].

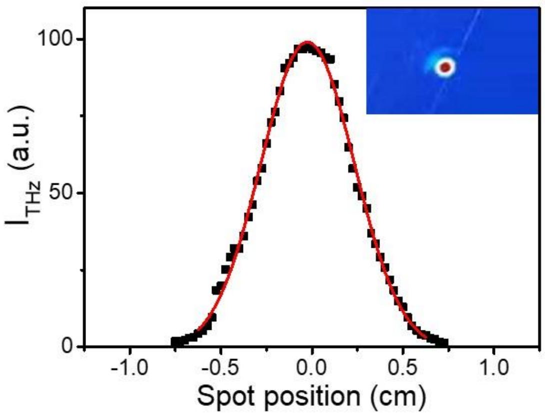

Supplementary Figure 10. 0.3 THz beam intensity profile along the vertical axis measured by the $NiTe_2$-based device. The inset shows a 0.3 THz beam image of the focused beam by using a THz camera.

Reference

1 Basko, D. A Photothermoelectric Effect in Graphene. *Science* **334**, 610-611 (2011).

2 El Fatimy, A., Myers-Ward, R. L., Boyd, A. K., Daniels, K. M., Gaskill, D. K. & Barbara, P. Epitaxial graphene quantum dots for high-performance terahertz bolometers. *Nat. Nano.* **11**, 335-338 (2016).

3 Lowy, T., Engely, M., Steiner, M. & Avouris, P. Origin of photoresponse in black phosphorus photo-transistors. *Phys. Rev. B* **90**, 081408 (2014).

4 E. Deyo, L.E.Golub, E.L. Ivchenko, B. Spivak. Semiclassical theory of the photogalvanic effect in non-centrosymmetric systems (2009).

5 Koniakhin, S. V. Ratchet effect in graphene with trigonal clusters. The European Physical Journal B 87, (2014).

6 Ferreira, A., Rappoport, T. G., Cazalilla, M. A. & Castro Neto, A. H. Extrinsic spin Hall effect induced by resonant skew scattering in graphene. *Phys. Rev. Lett.* 112, 066601, (2014).

7 Nalitov, A. V., Golub, L. E. & Ivchenko, E. L. Ratchet effects in two-dimensional systems with a lateral periodic potential. *Phys. Rev. B* 86, (2012).

8 W. Kohn, J. M. Luttinger, Quantum Theory of Electrical Transport Phenomena. *Phys. Rev.* 108, 590–611 (1957).

9 Park, C.-H., Yang, L., Son, Y.-W., Cohen, M. L. & Louie, S. G. Anisotropic behaviours of massless Dirac fermions in graphene under periodic potentials. *Nat. Phys.* 4, 213-217, (2008).

10 Trushin, M. & Schliemann, J. Anisotropic photoconductivity in graphene. *Euro. Phys. Lett.* 96, (2011).

11 Plank, H., Golub, L. E., Bauer, S., Bel'kov, V. V., Herrmann, T., Olbrich, P., Eschbach, M., Plucinski, L., Schneider, C. M., Kampmeier, J., Lanius, M., Mussler, G., Grützmacher, D. & Ganichev, S. D. Photon drag effect in$(Bi_{1-x}Sb_x)_2Te_3$ three-dimensional topological insulators. *Phys. Rev. B* **93** (2016).

12 Olbrich, P., Golub, L. E., Herrmann, T., Danilov, S. N., Plank, H., Bel'kov, V. V., Mussler, G., Weyrich, C., Schneider, C. M., Kampmeier, J., Grutzmacher, D., Plucinski, L., Eschbach, M. & Ganichev, S. D. Room-temperature high-frequency transport of dirac fermions in epitaxially grown Sb_2Te_3- and Bi_2Te_3-based topological insulators. *Phys. Rev. Lett.* **113**, 096601 (2014).

13 Sinova, J., Valenzuela, S. O., Wunderlich, J., Back, C. H. & Jungwirth, T. Spin Hall effects. *Reviews of Modern Physics* 87, 1213-1260, (2015).

14 E. N. Adams, E. I. Blount, Energy bands in the presence of an external force field–II: Anomalous velocities. *J. Phys. Chem. Solids* 10, 286–303 (1959).

15 L. Berger, Side-Jump Mechanism for the Hall Effect of Ferromagnets. *Phys. Rev. B* 2, 4559–4566 (1970).

16 N. A. Sinitsyn, Semiclassical theories of the anomalous Hall effect. *J. Phys. Cond. Matt.* 20, 023201 (2008).

17 Clark, O. J., Neat, M. J., Okawa, K., Bawden, L., Marković, I., Mazzola, F., Feng, J., Sunko, V., Riley, J. M., Meevasana, W., Fujii, J., Vobornik, I., Kim, T. K., Hoesch, M., Sasagawa, T., Wahl, P., Bahramy, M. S. & King, P. D. C. Fermiology and Superconductivity of Topological Surface States in $PdTe_2$. *Phys. Rev. Lett.* 120, (2018).

18 Song, J. C., Rudner, M. S., Marcus, C. M. & Levitov, L. S. Hot carrier transport and photocurrent response in graphene. *Nano Lett.* **11**, 4688-4692 (2011).
